# Comparison between D-loop methylation and mtDNA copy number in patients with Aicardi-Goutières Syndrome

**DOI:** 10.3389/fendo.2023.1152237

**Published:** 2023-03-14

**Authors:** Francesca Dragoni, Jessica Garau, Simona Orcesi, Costanza Varesio, Matteo Bordoni, Eveljn Scarian, Rosalinda Di Gerlando, Elisa Fazzi, Roberta Battini, Altea Gjurgjaj, Bartolo Rizzo, Orietta Pansarasa, Stella Gagliardi

**Affiliations:** ^1^ Department of Biology and Biotechnology “L. Spallanzani”, University of Pavia, Pavia, Italy; ^2^ Molecular Biology and Transcriptomics Unit, IRCCS Mondino Foundation, Pavia, Italy; ^3^ Neurogenetics Research Centre, IRCCS Mondino Foundation, Pavia, Italy; ^4^ Department of Brain and Behavioral Sciences, University of Pavia, Pavia, Italy; ^5^ Department of Child Neurology and Psychiatry, IRCCS Mondino Foundation, Pavia, Italy; ^6^ Cellular Model and Neuroepigenetics Unit, IRCCS Mondino Foundation, Pavia, Italy; ^7^ Department of Clinical and Experimental Sciences, University of Brescia, Brescia, Italy; ^8^ Unit of Child Neurology and Psychiatry, ASST Spedali Civili, Brescia, Italy; ^9^ Department of Developmental Neuroscience, IRCCS Stella Maris Foundation, Pisa, Italy; ^10^ Department of Clinical and Experimental Medicine, University of Pisa, Pisa, Italy

**Keywords:** Aicardi-Goutières Syndrome (AGS), methylation, mtDNA, D-loop (control region), epigenetics (DNA methylation), mitoepigenetics

## Abstract

**Introduction:**

Aicardi-Goutières Syndrome (AGS) is a rare encephalopathy with early onset that can be transmitted in both dominant and recessive forms. Its phenotypic covers a wide range of neurological and extraneurological symptoms. Nine genes that are all involved in nucleic acids (NAs) metabolism or signaling have so far been linked to the AGS phenotype. Recently, a link between autoimmune or neurodegenerative conditions and mitochondrial dysfunctions has been found. As part of the intricate system of epigenetic control, the mtDNA goes through various alterations. The displacement (D-loop) region represents one of the most methylated sites in the mtDNA. The term "mitoepigenetics" has been introduced as a result of increasing data suggesting that epigenetic processes may play a critical role in the control of mtDNA transcription and replication. Since we showed that RNASEH2B and RNASEH2A-mutated Lymphoblastoid Cell Lines (LCLs) derived from AGS patients had mitochondrial alterations, highlighting changes in the mtDNA content, the main objective of this study was to examine any potential methylation changes in the D-loop regulatory region of mitochondria and their relationship to the mtDNA copy number in peripheral blood cells of AGS patients with mutations in various AGS genes and healthy controls.

**Materials and methods:**

We collected blood samples from 25 AGS patients and we performed RT-qPCR to assess the mtDNA copy number and pyrosequencing to measure DNA methylation levels in the D-loop region.

**Results:**

Comparing AGS patients to healthy controls, D-loop methylation levels and mtDNA copy number increased significantly. We also observed that in AGS patients, the mtDNA copy number increased with age at sampling, but not the D-loop methylation levels, and there was no relationship between sex and mtDNA copy number. In addition, the D-loop methylation levels and mtDNA copy number in the AGS group showed a non-statistically significant positive relation.

**Conclusion:**

These findings, which contradict the evidence for an inverse relationship between D-loop methylation levels and mtDNA copy number, show that AGS patients have higher D-loop methylation levels than healthy control subjects. Additional research is needed to identify the function of these features in the etiology and course of AGS.

## 1 Introduction

Aicardi-Goutières Syndrome (AGS) is an early-onset and rare encephalopathy, inherited in a dominant and recessive form. Its phenotype includes a broad spectrum of neurological and extraneurological symptoms such as chronic cerebrospinal fluid (CSF) lymphocytosis, basal ganglia calcifications, leukodystrophy, and abnormal interferon-α release (1–3). Up to now, nine genes (*TREX1, RNASEH2B, RNASEH2A, RNASEH2C, ADAR1, SAMHD1, IFIH1, LSM11*, and *RNU7-1*), associated with AGS phenotype have been discovered, which are all involved in nucleic acids (NAs) metabolism or signaling (4–6). The majority of AGS patients carry mutations in the *RNASEH2A*, *RNASEH2B*, and *RNASEH2C* genes, which encode for the three subunits of the RNase H2 enzyme (4, 5, 7). The primary pathogenic hypothesis for AGS is the buildup of endogenous DNA or RNA: DNA hybrids that can activate an interferon (IFN)-α-mediated immune response (7). AGS is frequently included in the category of type I interferonopathies, a group of disorders characterized by an aberrant release of this kind of cytokines, as a result of the constitutive overexpression of type I interferons (8, 9).

A strong correlation between mitochondrial dysfunctions and autoimmune or neurodegenerative disorders has emerged in recent research (10, 11). Numerous autoimmune disorders are linked to elevated anti-mitochondrial antibodies and oxidative stress markers, supporting the idea that the mitochondria’s known role in the generation of reactive oxygen species (ROS) must be taken into account (12). Moreover there exist recent data underscoring the potential relationships between mitochondrial dysfunctions and interferon signaling (13).

The mitochondrial DNA (mtDNA) undergoes several modifications which are part of the complex system of epigenetic regulation. Numerous aspects of mitochondrial or neuronal function, inflammation and development are regulated by these mechanisms, such as DNA methylation, chromatin remodeling, and histone post-translational changes (14). Epigenetics, which can be studied at various depths, is primarily categorized into two sections: epigenetics of mitochondrial- and nuclear-encoded DNA. It has been demonstrated that mitochondrial epigenetic mechanisms have an impact on diseases, cell destiny, transcription regulation, cell division, and cell cycle (15). In this context, the term “mitoepigenetics” has been introduced as a result of increasing data suggesting that epigenetic processes may play a critical role in the control of mtDNA transcription and replication (16). Nowadays, it is evident that the levels of methylation and hydroxymethylation in mtDNA are significantly lower than those in nuclear DNA (nDNA), however, changes in DNA methylation and hydroxymethylation occur in the mtDNA (17) and regulate both mtDNA replication and gene expression levels. Among the mtDNA sites, the displacement (D-loop) region represents one of the most methylated sites in the mtDNA, with average methylation levels of less or about 5% (17, 18). Human and mouse cell cultures (19, 20), peripheral blood cells (21–25), colorectal cancer tissues (26), the human placenta (27), as well as, mesenchymal stem cells (28), and colorectal cancer tissues (26, 29) have all shown a correlation between mtDNA methylation and gene expression (22).

Since we demonstrated the presence of mitochondrial alterations in *RNASEH2B* and *RNASEH2A*-mutated Lymphoblastoid Cell Lines (LCLs) derived from AGS patients, highlighting alterations in the mtDNA content (30), the aim of this study was to assess the possible presence of methylation changes in the D-loop regulatory region of mitochondria and its relation with the mtDNA copy number in peripheral blood cells of AGS patients carrying mutations in different AGS genes and healthy controls as a possible pathomechanism in AGS.

## 2 Materials and methods

### 2.1 Patients enrollment

Blood samples from 25 AGS patients (12 females and 13 males) carrying different AGS-related mutations were collected at Child and Adolescent Neurology Unit of the IRCCS Mondino Foundation (Pavia, Italy) in EDTA tubes. Blood samples from 22 healthy volunteers (12 females and 10 males), free of any pharmacological therapy or pathology, were collected at the Immunohematological and Transfusional Service of the Fondazione IRCCS Policlinico San Matteo (Pavia, Italy). Age and sex of patients and healthy controls are reported in **Table 1** and **Supplementary S1**. Mutations for each patient are listed in **Table 2**. Being the most commonly altered gene among AGS patients, our cohort was composed prevalently by *RNASEH2B* p.A177T mutated patients who showed mild to severe symptoms. This allowed us to correlate our results with the phenotypic features.

**Table 1:** Study subjects.

Group	Sex (F/M)	Average Age
Control subjects n=22	12/10	27
AGS patients n=25	12/13	6

**Table 2:** Gene mutations.

Number of patients	Gene	Mutation
n=12	*RNASEH2B*	p.A177T
n=1	*RNASEH2B*	p.A177T+p.T163I
n=1	*RNASEH2B*	p.A177T+ p.A212V
n=1	*RNASEH2B*	p.A177T+c.436+1 G>T
n=1	*RNASEH2B*	p.V185G
n=1	*RNASEH2A*	p.R108W+p.F230L
n=4	*SAMHD1*	p.M385V
n=2	*ADAR1*	p.P193A *+* c.1076_1080del
n=1	*TREX1*	p.R169H+p.V290fs
n=1	*IFIH1*	p.R779C

### 2.2 DNA extraction

Genomic DNA extraction was performed using a semi-automated method Maxwell^®^ 16 System DNA Purification (Promega, Madison, WI, USA). DNA was quantified with NanoDrop ND1000 UV-Vis Spectrophotometer and Qubit^®^ fluorometer (Thermo Scientific, Waltham, MA, USA).

### 2.3 mtDNA copy number quantification

10 ng of total cellular DNA were used as the input for semi-quantitative PCR (qPCR) to measure the number of mtDNA copies. **Table 3** lists the primers used to amplify a nuclear DNA (nDNA) region (hemoglobin subunit beta, HBB) and an mtDNA region (chrM: 3, 313-3,322) (31, 32), respectively. 200 nM of each oligonucleotide (Metabion, Planegg/Steinkirchen, Germany), 7.5 μL of SYBR Green SuperMix (BioRad, Richmond, CA, USA), and 1 μL of DNA template (10 ng/μL) or water control were used in qPCR experiments. Each repeat qPCR experiment had its cycle threshold (Ct) values automatically recorded, and the mean Ct values were standardized to those found for the HBB gene.

**Table 3:** Primers sequences for mtDNA copy number analysis.

Region	Primer Forward	Primer Reverse
***mtDNA***	CACCCAAGAACAGGGTTTGT	TGGCCATGGGTATGTTGTTA
***Hemoglobin subunit β (HBB)***	GCTTCTGACACAACTGTGTTCACTAGC	CACCAACTTCATCCACGTTCACC

To determine the mtDNA content relative to nDNA, the following equations were used (33):


(1) ΔCt=nDNA Ct−mtDNA Ct

(2) Relative mtDNA content=2×2ΔCt

### 2.4 D-loop methylation analysis

To turn all unmethylated cytosines into uracil, 500 ng of DNA from each sample were exposed to sodium bisulfite treatment. The EpiTect Bisulfite Kit (Qiagen, Hilden, Germany) was used to convert the bisulfite according to the manufacturer’s instructions. In the Heavy (H)-strand of the D-loop region, ranging from nucleotides 16,417 to nucleotides 73 in mtDNA, three distinct CpG sites were employed to measure DNA methylation using bisulfite pyrosequencing (GenBank: J01415.2). Using primers from published research (32), one amplicon (226 bp) was produced. The following parts were used in a 25 μL PCR: 12 μL of PyroMark PCR Master Mix 2x, 2.5 μL of CoralLoad Concentrate 10x, 2 μL of forward and primer mix (2,5 μM), RNase-free water (variable), and 10 ng of bisulfite converted DNA (Qiagen, Hilden, Germany). The following procedure was used for the PCR amplifications: 15 minutes at 95°C, then 45 cycles of 30 seconds each at 94°C, 56°C, and 72°C, with a final extension of 10 minutes at 72°C. Using the sequencing primer, pyrosequencing was used to purify and sequence the PCR products. DNA methylation levels were measured using the PyroMark Q48 Adv. CpG Reagents (Qiagen, Hilden, Germany), PyroMark Q48 Autoprep 4.2.1 software, and the manufacturer’s recommended procedures. The percentage of 5-methylcytosine is used to represent the methylation level (5-mC). The mean methylation levels of the first three CpG sites in the amplicon were used for the analysis.

### 2.5 Statistical analysis

Statistical analysis were performed and figures were obtained with GraphPad PRISM version 9 (San Diego, CA, USA). Pearson’ correlation coefficients were used to evaluate correlations between D-loop methylation levels, mtDNA copy number, and age at sampling. mtDNA copy number and D-loop methylation levels were compared between groups by means of Student’s t test and data are presented as mean ± SEM. When the p-values were less than 0.05, the results were considered statistically significant.

## 3 Results

A total of 47 subjects were involved in the current study, including 25 AGS patients and 22 healthy controls (**Tables 1**, **2**). Since the *RNASEH2B* is the most commonly altered gene in AGS, in this work, the AGS group was composed of sixteen *RNASEH2B*-mutated patients, twelve of whom carry the p.A177T mutation with both mild and severe phenotypes, one *RNASEH2A*, *TREX1* and *IFIH1*-mutated patients, four patients carrying mutations in *SAMHD1* gene and two ADAR1-mutated patients. qPCR was used to assess the mtDNA copy number and bisulfite pyrosequencing was performed to measure DNA methylation levels in the mitochondrial D-loop region.

### 3.1 Analysis of mtDNA copy number and D-loop methylation levels in AGS patients and healthy controls

**Figure 1:**
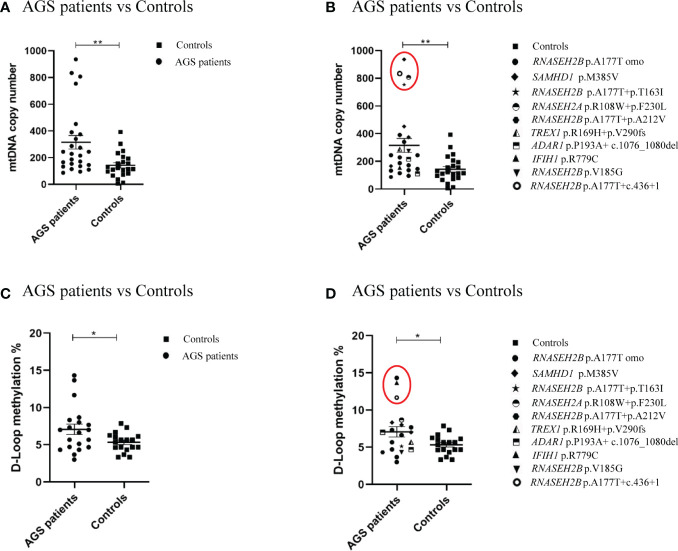
**(A)** mtDNA copy number in the healthy control group (n=22), in AGS patients (n=25) and **(B)** AGS patients stratified on gene mutations (*RNASEH2B* p.A177T n=12; *RNASEH2B* p.A177T+p.T163I n=1; *RNASEH2B* p.A177T+p.A212V n=1; *RNASEH2B* p.V185G n=1; RNASEH2B p.A177T+c.436+1 n=1; *RNASEH2A* p.R108W+p.F230L n=1; *SAMHD1* p.M385V n=4; *ADAR1* p.P193A+ c.1076_1080del n=2; *TREX1* p.R169H+p.V290fs n=1; *IFIH1* (p.R779C) n=1. **(C)** D-loop methylation in the healthy control group (n=18), AGS patients (n=20) and **(D)** AGS patients stratified on gene mutations (*RNASEH2B* p.A177T n=8; *RNASEH2B* p.A177T+p.T163I n=1; *RNASEH2B* p.A177T+p.A212V n=1; *RNASEH2B* p.V185G n=1; *RNASEH2B* p.A177T+c.436+1 n=1; *RNASEH2A* p.R108W+p.F230L n=1; *SAMHD1* p.M385V n=3; *ADAR1* p.P193A+ c.1076_1080del n=2; *TREX1* p.R169H+p.V290fs n=1; *IFIH1* p.R779C n=1). **(B, D)** Red circles highlights the gene mutations which drive the differences. Statistical analysis was performed using the Student’s t test and data are expressed by means ± SEM; *p< 0.05; **p< 0.01.

The comparison between mtDNA copy number and D-loop methylation levels in AGS patients and healthy controls groups is shown in **Figure 1**. The mtDNA copy number was significantly higher in AGS patients compared to the healthy controls group (-172.9 ± 56.68; p=0.0038; 95% CI=-287.1 to -58.78) (**Figure 1A**). The difference was driven by two *RNASEH2B*-mutated patients carrying different mutations (*RNASEH2B* p.A177+ c.436+1, *RNASEH2B* p.A177T + p.T163I), one *RNASEH2A*-mutated patient and one *SAMHD1*-mutated patient (**Figure 1B**). The D-loop methylation level was significantly higher in AGS patients compared to healthy controls (**Figure 1C**) as well (-1.747 ± 0.7909; p=0.0168; 95% CI=-3.351 to -0.1429), and the difference was driven by two *RNASEH2B*-mutated patients carrying the same p.A177T mutation and the *IFIH1*-mutated patient (**Figure 1D**).

**Figure 2:**
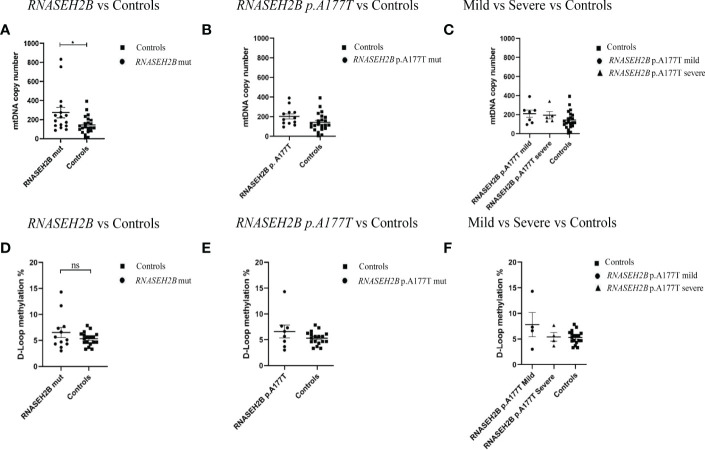
**(A)** mtDNA copy number in the healthy control group (n=22) and in AGS *RNASEH2B*-mutated patients (n=16); **(B)** in *RNASEH2B* p.A177T-mutated patients (n=12) and **(C)** *RNASEH2B* p.A177T-mutated mild and severe patients (n=7 and n=5, respectively). **(D)** D-loop methylation in the healthy control group (n=18), and AGS *RNASEH2B*-mutated patients (n=12); **(E)** in *RNASEH2B* p.A177T- mutated patients (n=8) and **(F)** *RNASEH2B* p.A177T-mutated mild and severe patients (n=4 and n=4, respectively). Statistical analysis was performed using the Student’s t test and data are expressed by means ± SEM. *p< 0.05. ns, not significant.

Since *RNASEH2B* is the most commonly altered gene in AGS patients and, in particular, p.A177T is the most common mutation found in *RNASEH2B*-mutated patients, we sought to compare mtDNA copy number and the methylation level of the D-loop region by plotting only the *RNASEH2B*-patients. The same mutation, p.A177T, is associated with two different phenotypes (mild and severe) (34) and along with typically severe phenotypes, this specific mutation is among the most commonly associated with later onset and milder manifestations, with some subjects having relatively preserved intellectual function, communication skills and manual abilities. Starting from these assumptions we tried to assess whether there could be a difference between patients with mild and severe AGS phenotype in terms of mtDNA content and D-loop region methylation levels (**Figure 2**). We found that, taking into account only the *RNASEH2B*-mutated patients, the mtDNA copy number remains significantly higher when compared to healthy controls (-131.4 ± 52.36; p=0.0168; 95% CI= -237.6 to -25.18). Specifically, two *RNASEH2B* patients are responsible for the difference (**Figure 2A**). Plotting data for *RNASEH2B* p.A177T-mutated patients (-60.23 ± 33.26; p=0.0796; 95% CI=-128.0 to 7.528) emerged that none of them drove the significant variance. The two patients responsible for the changes carried the *RNASEH2B* p.A177T+p.T163I mutation and the *RNASEH2B* p.A177T+c.436+1 mutation (**Figures 2A, B**). These data suggest that the highest mtDNA content is associated with a specific gene but not associated with the same specific mutation. We did not find a particular correlation with the phenotype disparities in fact, deeping into the *RNASEH2B* p.A177T mutation, we did not observe any differences in the mtDNA between mild (-66.74 ± 41.34; p= 0.1181; 95% CI= -151.6 to 18. 08) and severe phenotype (-51.10 ± 45.67; p= 0.2738; 95% CI= -145.2 to 42.95) when compared to healthy controls (**Figure 2C**).

Accordingly, the comparison of the D-loop methylation levels between *RNASEH2B*-mutated AGS patients and healthy controls (**Figure 2D**) showed that the methylation range was higher, but not statistically significant, in the AGS group (-1.212 ± 0.8730; p=0.1759; 95% CI= -3.000 to 0.5760). Following the approach applied for the mtDNA copy number, we plotted only the *RNASEH2B* p.A177T mutated patients-derived data (**Figure 2E**) and found that the D-loop methylation was still higher when compared to healthy controls and the difference was driven by one patient carrying the *RNASEH2B* p.A177T mutation (-1.299 ± 0.9324; p=0.1762; 95% CI= -3.224 to 0.6250). Besides, this difference was associated with a mild phenotype (-2.496 ± 1.207; p=0.0519; 95% CI= -5.015 to 0.02218) although the divergence was not statistically significant (**Figure 2F**).

**Figure 3:**
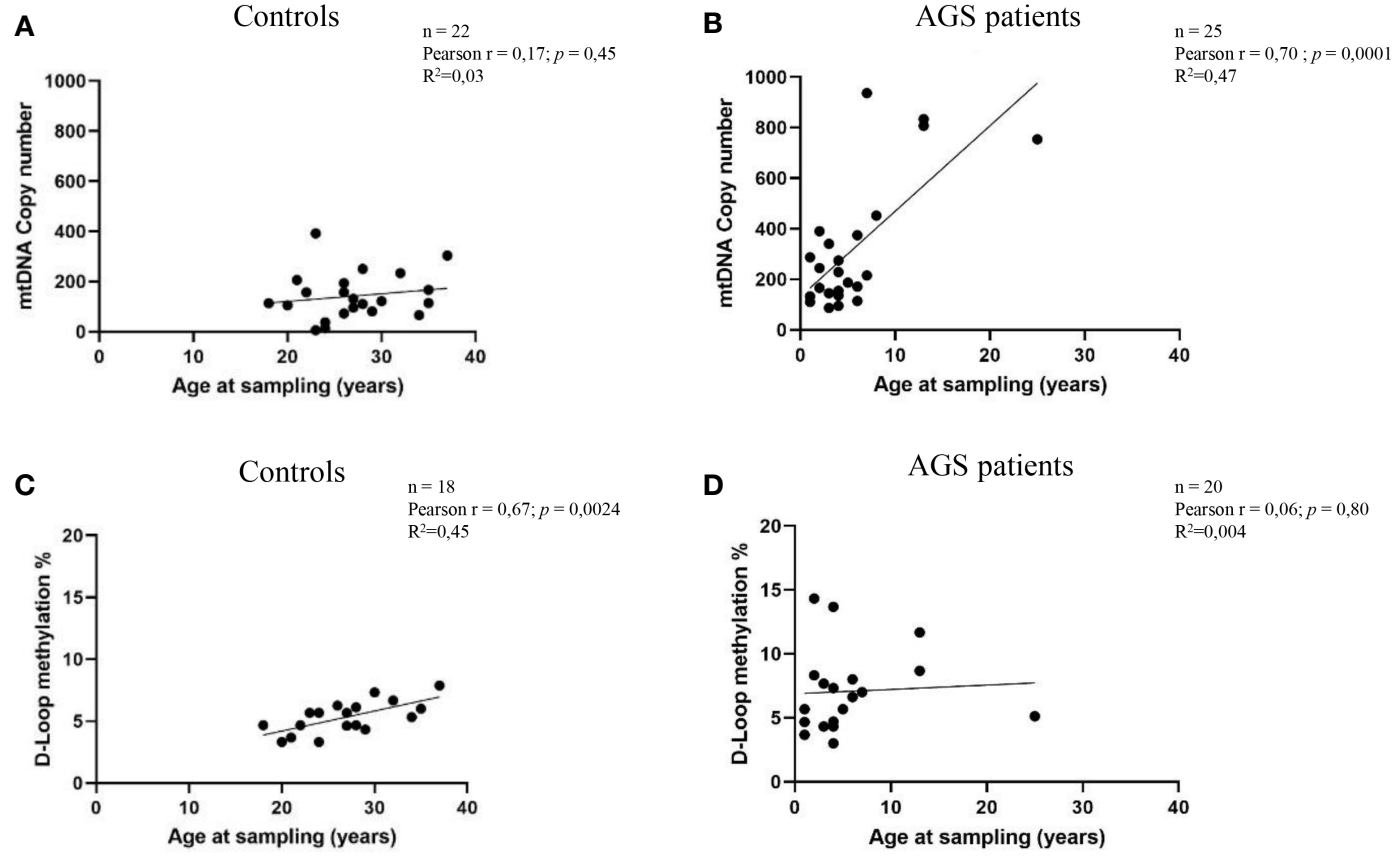
**(A)** Correlation between age at sampling and mtDNA copy number in the healthy control group (n=22) **(B)** and AGS patients (n=25). **(C)** Age at sampling and the levels of D-loop methylation were correlated in the control group (n=18) and **(D)** AGS patients (n=20). Pearson’s correlation coefficient and R square (R^2^) were used to examine the relationship between age at sampling and mtDNA copy number or D-loop methylation levels.

**Figure 3** depicts how mtDNA copy number and D-loop methylation levels are affected by the age at sampling. A positive correlation was detected between AGS patients’ mtDNA copy number and the age of sampling (Pearson r=0.70; R^2 =^ 0.47; p=0.0001; 95% CI=- 0.4179 to 0.8567) as well as in controls subjects (Pearson r=0.17; R^2 =^ 0.03; p=0.45; 95% CI=-0.2708 to 0.5522) (**Figures 3A, B**). This correlation was significant only in AGS patients. Conversely, a positive but not significant correlation emerged between AGS patients (Pearson r=0.06; R^2 =^ 0.004; p=0.80; 95% CI=-0.3916 to 0.4908) or healthy controls (Pearson r=0.67; R^2 =^ 0.45; p=0.0024; 95% CI=0.2940 to 0.8656) and D-loop methylation levels (**Figures 3C, D**).

**Figure 4:**
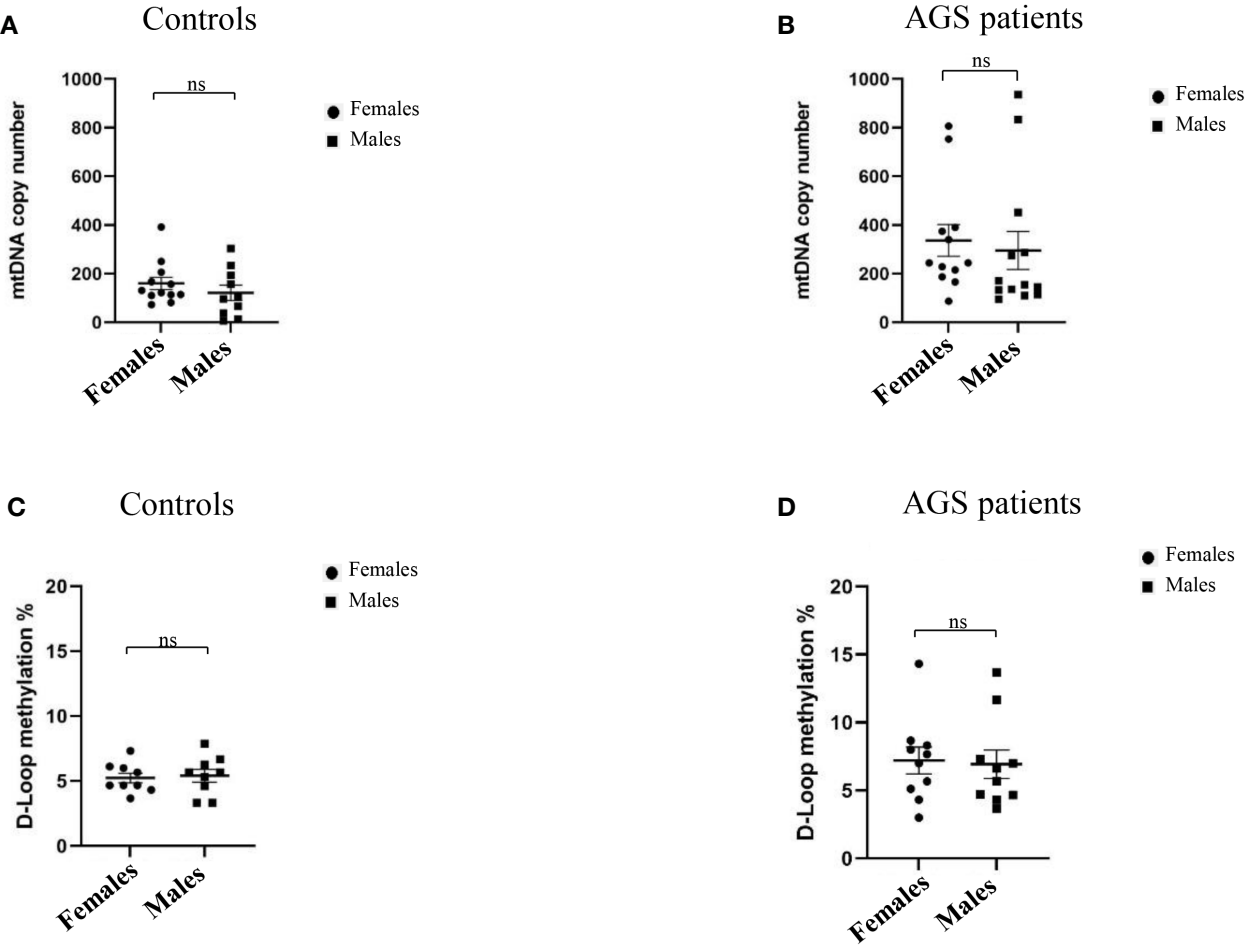
**(A)** Effect of sex on mtDNA copy number in the healthy control group (n=22) and **(B)** in AGS patients (n=25). Effect of sex on the levels of D-loop methylation in **(C)** the healthy control group (n=18), and **(D)** AGS patients (n=20). Statistical analysis was performed using the Student’s t test and data are expressed by means ± SEM. ns, not significant.

Moreover, **Figure 4** highlights how sex could modulate the mtDNA copy number and D-loop methylation levels of AGS patients and healthy controls. We did not find any significant difference in mtDNA copy number between males and females in both healthy controls (-38.51 ± 40.07; p=0.3481; 95% CI=-122.1 to 45.09) and AGS patients (-40.92 ± 102.2; p=0.6925; 95% CI=-40.92 ± 102.2) (**Figures 4A, B**). Also, the D-loop methylation level was not significantly affected by sex in both healthy controls (0.1804 ± 0.6230; p=0.3879; 95% CI=-1.140 to 1.501) and AGS patients (-0.2785 ± 1.433; p=0.4240; 95% CI=-3.289 to 2.732) as well (**Figures 4C, D**).

**Figure 5:**
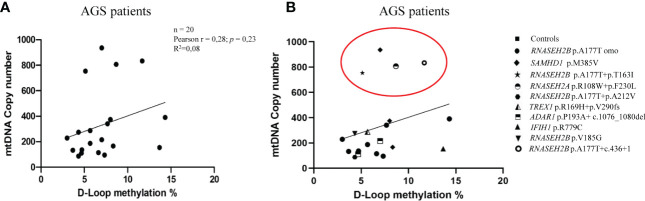
**(A)** Correlation between mtDNA copy number and D-loop methylation levels in the AGS patients group (n=20). **(B)** Stratification of AGS patients on genes mutation (RNASEH2B p.A177T n=9; RNASEH2B p.A177T+p.T163I n=1; RNASEH2B p.A177T+p.A212V n=1; RNASEH2B p.V185G n=1; RNASEH2B p.A177T+c.436+1 n=1; RNASEH2A p.R108W+p.F230L n=1; SAMHD1 p.M385V n=3; ADAR1 p.P193A+c.1076_1080del n=2; TREX1 p.R169H+p.V290fs n=1; IFIH1 p.R779C n=1). Red circle highlights the gene mutations which drive the differences. Pearson's correlation coefficient and R square (R2) were used to examine the relationship between mtDNA copy number and D-loop methylation levels in AGS patients.

In **Figure 5**, we directly correlated the mtDNA copy number with the D-loop methylation levels in AGS patients and controls groups. We found a positive, but not significant, correlation AGS patients group (Pearson r= 0.2783; R^2 =^ 0.08; p=0.2347; 95% CI= -0.1874 to 0.6419) (**Figures 5A, B**).

## 4 Discussion

The AGS is a rare hereditary encephalopathy that typically appears within the first year of life. It is inherited in both autosomal recessive and dominant forms. All AGS-related genes encode for proteins involved in the DNA damage response and NAs metabolism, and their mutations may result in a mishandled innate immune response, leading to increased IFN-α production (4, 35). The RNAse H2 protein, composed by three different subunits, *RNASEH2A*, *RNASEH2B*, and *RNASEH2C* (4, 5, 7), may be involved in a variety of processes related to NAs metabolism, and it is the main source of cellular ribonuclease activity in eukaryotes (36). In the last years, relevance of mitochondrial dysfunction in autoimmune and inflammatory illnesses has been shown (11, 12), with a strong correlation between the mtDNA release and the induction of an abnormal IFN-α-dependent immune response (37, 38). Furthermore, many studies have focused on the investigation of mitochondrial epigenetics as well as on the central function of mitochondria in controlling cell metabolism (39, 40). In blood and brain samples collected from patients and from animal’s models of neurodegeneration, several authors (19, 20, 26, 39, 40) looked for variations in mtDNA methylation. The heavy mtDNA strand’s origin of replication is located in the 1.1 kb non-coding mitochondrial region known as D-loop and it is essential for mitochondrial replication, transcription and a promising target for epigenetic changes (19). The mtDNA D-loop has been discovered to be methylated at both CpG and non-CpG locations, although the underlying molecular mechanisms are still unknown (41).

In the current work we aimed, for the first time, to broaden our knowledge on the relation between mtDNA copy number and D-loop methylation levels in AGS patients. We examined mtDNA copy number and D-loop methylation levels in peripheral blood cells from 47 samples, including 25 AGS patients and 22 healthy controls. D-loop methylation levels and mtDNA copy number showed a statistically significant increase in AGS patients compared to healthy control subjects. These differences were associated prevalently with the *RNASEH2B* mutations, the most commonly altered gene in the pathology (4, 5). These results are in accordance with our previously published data which underlined an increase in the mtDNA copy number particularly in *RNASEH2B*-mutated LCLs (30), but further investigations are needed to better understand how the mtDNA methylation influences the mtDNA copy number increase. Nothing was previously established or described in the literature in relation to this condition, so we explored this feature as well as the pattern of methylation in mtDNA for the first time. According to our hypothesis, the amount of mtDNA may be higher in AGS patients and may be released from the abnormal mitochondria, activating immune systems, and producing the elevated levels of IFN- α that are typical of the illness. Interferon stimulated genes (ISGs) are more significantly expressed in the LCLs of AGS patients, as described in another study of ours (30) which also demonstrated the overexpression of the TLR9 gene. Moreover, although we are aware that the age difference between healthy controls and AGS patients, may be a limitation in the correlation, we also observed that the mtDNA copy number, but not the D-loop methylation levels, increased with increasing age at sampling in AGS patients but there was no correlation with sex. Since AGS is a disease that affects very young children, it does not allow us to obtain healthy controls of a comparable age, but subjects of an age as close as possible to the affected patients were included in our study. Furthemore, a non-statistically significant positive correlation between D-loop methylation levels and the mtDNA copy number was observed in AGS group. The action of 5-mC is typically thought to interfere with transcriptional start and suppress gene expression (42, 43) and an inverse correlation between D-loop methylation levels and the mtDNA copy number has been reported in literature several times in human and mouse cell cultures, in human peripheral blood cells, in cancer tissues and human placenta too (17, 19, 20, 32). Supporting evidences derive from the assumption that the D-loop region is a plausible candidate for epigenetic alterations since it enables mtDNA replication by preserving an open structure of the DNA (19, 44). Accordingly, DNA methyltransferase 1 (DNMT1), the enzyme in charge of maintaining DNA methylation, can attach to the mitochondrial D-loop area and cause transcription of mtDNA genes to be repressed, hence lowering the number of copies of mtDNA. However, Byun and collaborators reported that the mtDNA methylation was positively correlated with mtDNA copy number (21). To date, it is known that changes in methylation distribution can affect the same area in different diseases or cell types (39, 40). Additionally, in our previous research (30), we demonstrated that the rates of oxidative phosphorylation and ROS production are elevated in AGS patients, which suggests that they may be a contributing factor in the oxidative stress condition we demonstrated to be present, particularly in *RNASEH2B*-mutated patients. More research is required to determine how these factors, along with an increase in mtDNA copy number and the activation of inflammatory pathways, may contribute to some of the symptoms experienced by AGS patients.

In conclusion, this study reveals a higher D-loop methylation levels in AGS patients than in healthy control subjects and counteracts the evidences of an inverse correlation between D-loop methylation levels and mtDNA copy number. Further studies are required to better understand the methylation distribution pattern in AGS patients, also considering different mtDNA regions, and to define the role of these features in the AGS etiology and in disease course (for example how external factors, such as infection or immune stimulation with vaccination, can affect the immune response).

## Data availability statement

The datasets presented in this study can be found in online repositories. The names of the repository/repositories and accession number(s) can be found in the article/**Supplementary Material**.

## Ethics statement

The studies involving human participants were reviewed and approved by IRCCS Mondino Pavia: CE Pavia; IRCCS Fondazione Stella Maris: CE Pediatrico Regionale and ASST Spedali Civili Brescia: Ethics CE di Brescia (Protocol n°375/04 of 07/01/2004; n° 3549/2009 of 30/9/2009 and 11/12/2009, and n°20170035275 of 23/10/2017). Written informed consent was obtained from the individuals and/or their legal guardian/next of kin for participation in the study and the publication of any potentially identifiable data included in the article and/or supplementary material.

## Author contributions

Conceptualization: FD, JG, SO, CV, OP, and SG. Formal analysis: FD, JG, MB, ES, RD, OP, and SG. Funding acquisition: SO, OP, and SG. Investigation: FD, JG, AG, BR, OP, and SG. Methodology: FD, JG, AG, BR, OP, and SG. Resources: FD, JG, SO, CV, OP, and SG. Supervision: OP and SG. Visualization: FD, JG, AG, BR, OP, and SG. Writing—original draft: FD, JG, SO, CV, MB, ES, RD, EF, BR, OP, and SG. Writing—review and editing: FD, JG, SO, CV, MB, ES, RD, EF, BR, OP, and SG. All authors contributed to the article and approved the submitted version.
