# Supplementary material for: Comparison between D-loop methylation and mtDNA copy number in patients with Aicardi-Goutières Syndrome
Source: Front Endocrinol (Lausanne). 2023 Mar 14;14:1152237. doi: 10.3389/fendo.2023.1152237 (PMC10043473; doi:10.3389/fendo.2023.1152237)
Supplement: Supplementary file 1 [file Table_1.docx]

Supplementary Material

Comparison between D-Loop methylation and mtDNA copy number in patients with Aicardi-Goutières Syndrome

**Francesca Dragoni1^2†^, Jessica Garau^3†^, Simona Orcesi^4,5^, Costanza Varesio^4,5^, Matteo Bordoni^6^, Eveljn Scarian^4,6^, Rosalinda Di Gerlando^1,2^, Elisa Fazzi^7^, Roberta Battini^8,9^, Altea Gjurgjaj^1^, Bartolo Rizzo^1^, Orietta Pansarasa^6*^, Stella Gagliardi^2§^**

*** Correspondence:** Orietta Pansarasa; [**orietta.pansarasa@mondino.it**](mailto:orietta.pansarasa@mondino.it)

| **Sample** | **Age** | **Sex** |
| --- | --- | --- |
| AGS 1 | 4 | F |
| AGS 2 | 2 | F |
| AGS 3 | 1 | M |
| AGS 4 | 1 | M |
| AGS 5 | 3 | F |
| AGS 6 | 2 | F |
| AGS 7 | 1 | M |
| AGS 8 | 3 | F |
| AGS 9 | 4 | M |
| AGS 10 | 5 | F |
| AGS 11 | 4 | M |
| AGS 12 | 13 | F |
| AGS 13 | 7 | M |
| AGS 14 | 4 | M |
| AGS 15 | 6 | F |
| AGS 16 | 7 | F |
| AGS 17 | 13 | M |
| AGS 18 | 4 | M |
| AGS 19 | 6 | M |
| AGS 20 | 25 | F |
| AGS 21 | 3 | M |
| AGS 22 | 2 | F |
| AGS 23 | 2 | M |
| AGS 24 | 1 | F |
| AGS 25 | 8 | M |
| HC 1 | 28 | F |
| HC 2 | 20 | M |
| HC 3 | 18 | F |
| HC 4 | 27 | F |
| HC 5 | 22 | F |
| HC 6 | 24 | M |
| HC 7 | 21 | F |
| HC 8 | 24 | M |
| HC 9 | 23 | M |
| HC 10 | 32 | M |
| HC 11 | 34 | M |
| HC 12 | 35 | F |
| HC 13 | 30 | F |
| HC 14 | 29 | F |
| HC 15 | 26 | M |
| HC 16 | 27 | M |
| HC 17 | 37 | M |
| HC 18 | 28 | F |
| HC 19 | 26 | F |
| HC 20 | 23 | F |
| HC 21 | 26 | M |
| HC 22 | 35 | F |

**Supplementary S1.** Age and sex of all AGS patients and Healthy controls involved in the study. Age: Age at sampling; AGS:AGS patients; HC: Healthy control.
